# Supplementary material for: The Candidate IBD Risk Gene CCNY Is Dispensable for Intestinal Epithelial Homeostasis
Source: Cells. 2021 Sep 6;10(9):2330. doi: 10.3390/cells10092330 (PMC8471355; doi:10.3390/cells10092330)
Supplement: Supplementary file 1 [file cells-10-02330-s001.zip › cells-1245389-supplementary.pdf]

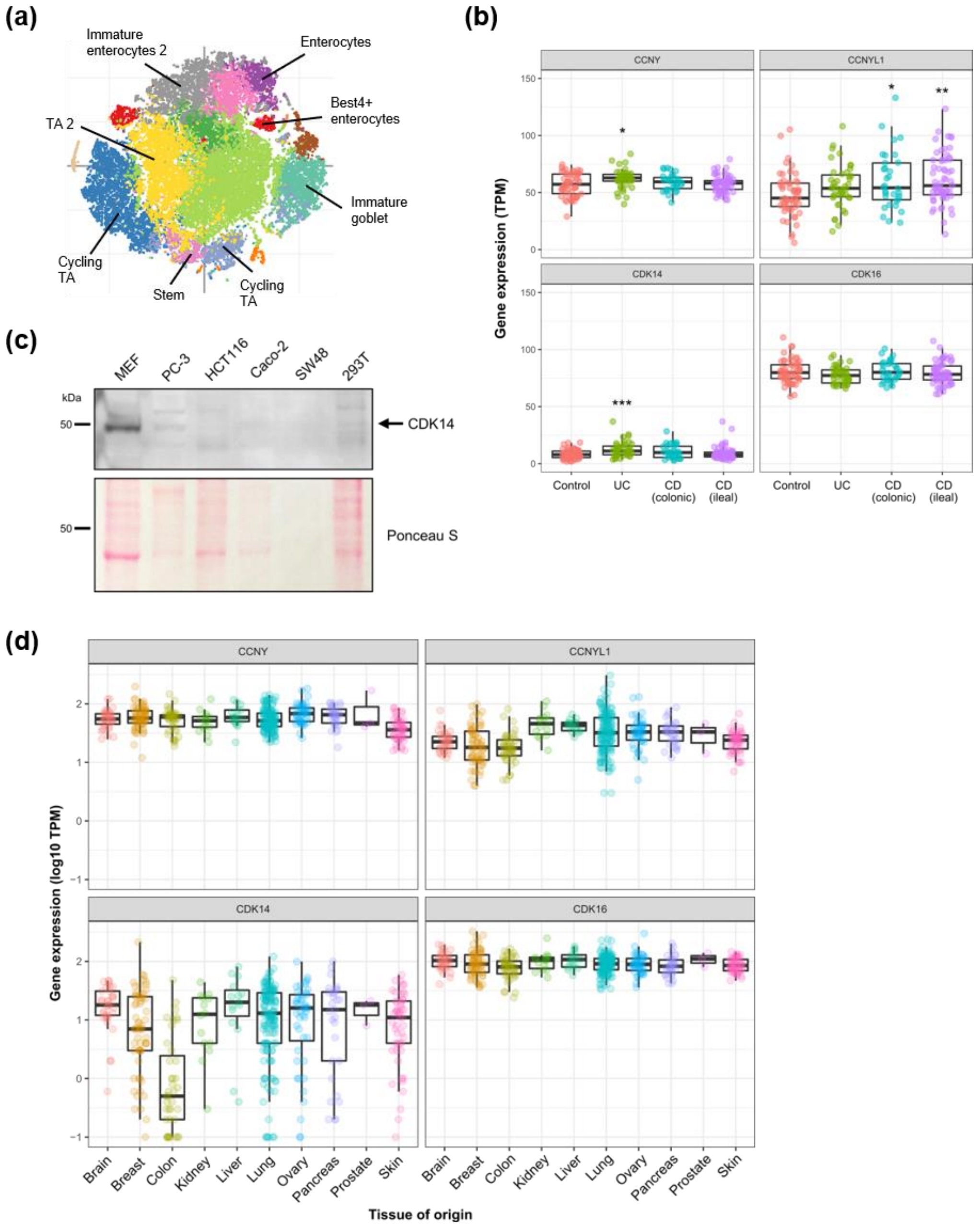


**Figure S1:** Expression analysis of *CCNY/L1* and *CDK14/16*. (**a**) Cluster annotation of the t-SNE plots shown in Figure 1, Single Cell Portal dataset SCP259. Only the most relevant cell populations are highlighted using the original author annotations. (**b**) RNA-seq gene expression analysis of rectal biopsies from pediatric IBD patients, GEO dataset GSE117993. *CCNY/L1* and *CDK14* expression were modestly increased in ulcerative colitis (UC) and Crohn's disease (CD) samples, but *CDK14* levels were generally low. * $p<0.05$, ** $p<0.01$, and *** $p<0.001$ *versus* Control (Dunnett's post-hoc test following one-way ANOVA). (**c**) Immunoblot of CDK14. A band of the expected molecular weight was detected in mouse embryonic fibroblasts (MEF), prostate adenocarcinoma cells (PC-3), and, to a lesser extent, human embryonic kidney cells (293T), but not the colorectal cancer (CRC) cell lines HCT116 and Caco-2. Results for SW48 CRC cells were inconclusive due to low protein content. Bulk proteins were detected by Ponceau S staining after transfer. (**d**) RNA-seq gene expression analysis of cancer cell lines, EMBL-EBI dataset E-MTAB-2706. Each dot represents one cell line derived from the indicated tissue of origin.

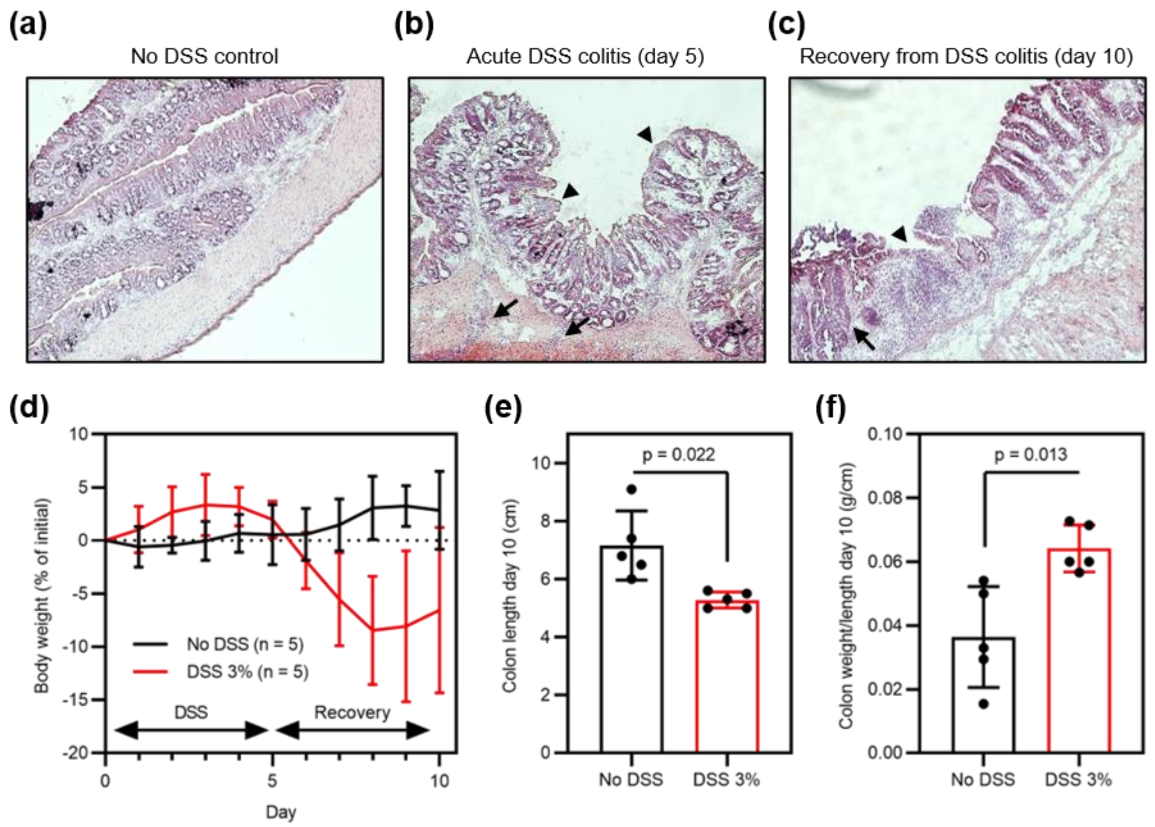


**Figure S2:** Assessment of DSS colitis disease activity. (**a-c**) Representative H&E images of colon sections from wild-type mice treated, where indicated, with 3% DSS for 5 days. Images were taken at 10x original magnification. In (**b**), note mucosal leukocyte infiltration (arrows) and epithelial damage (arrowheads) characteristic for acute DSS colitis. In (**c**), note crypt fission (arrow) and re-epithelization (arrowhead) indicative of tissue regeneration. (**d**) Body weight change in wild-type mice treated with 3% DSS as indicated. (**e, f**) Colon length and colon weight/length ratio of the animals from panel (**d**). Data were analyzed using Welch's unpaired t-test.

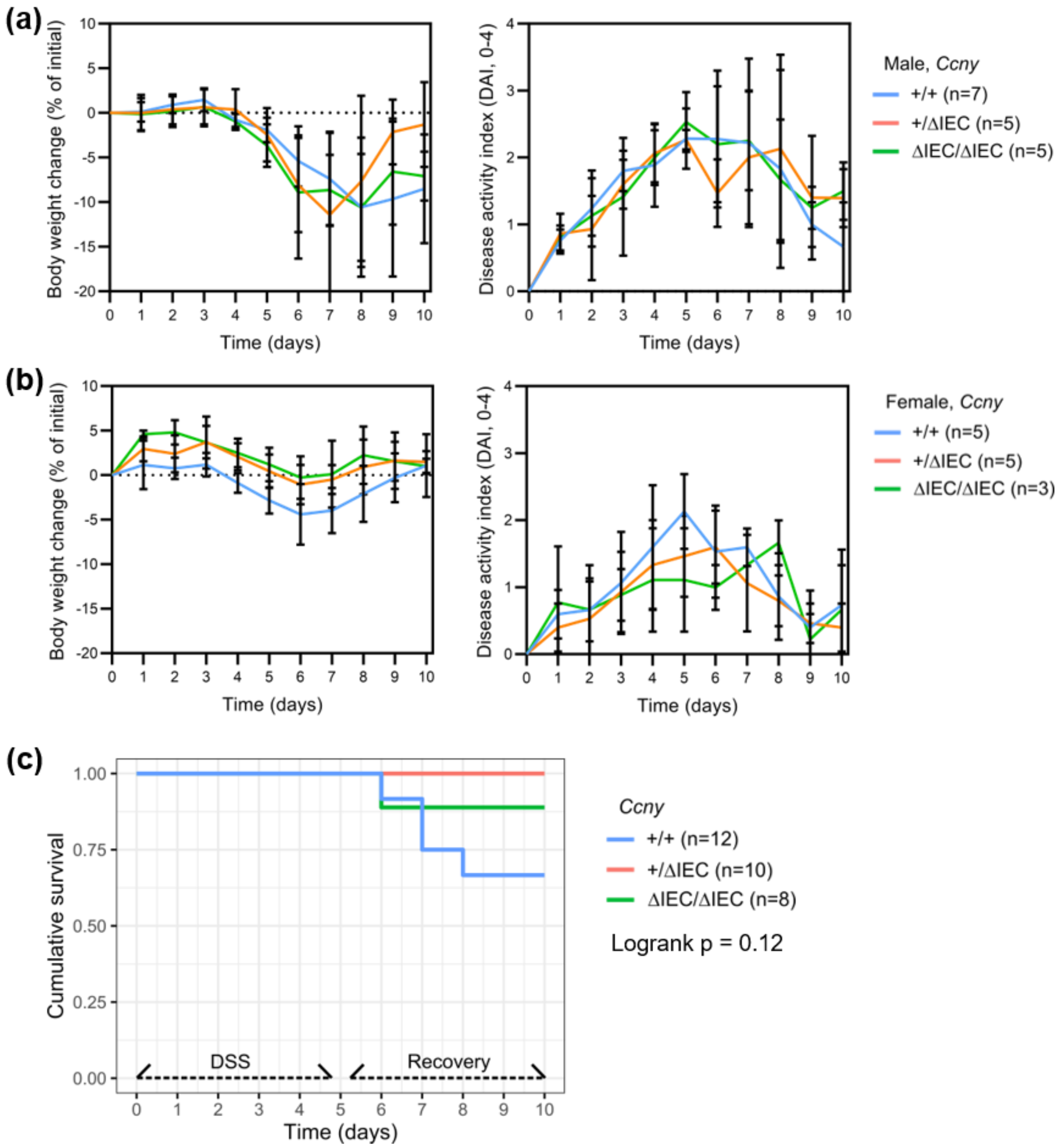


**Figure S3:** Loss of intestinal epithelial *Ccny* does not affect the susceptibility to DSS colitis. (**a, b**) Body weight change and disease activity of male (**a**) and female (**b**) *Ccny*$^{\Delta IEC}$ mice during the recovery from dextran sulfate sodium (DSS)-induced colitis. No difference was observed compared to littermate controls. (**c**) Kaplan-Meier survival plot of mice that were removed from the experiment at an ethical endpoint.

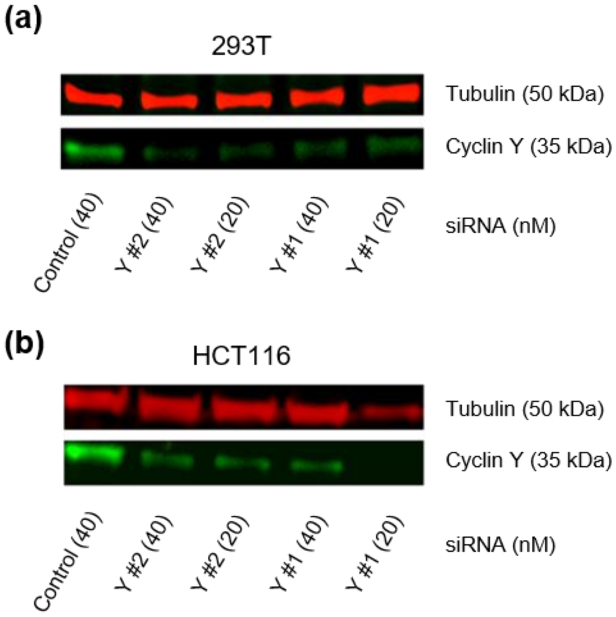


**Figure S4:** *CCNY* siRNA and antibody validation. (**a**, **b**) Cyclin Y loss-of-function was assessed by RNA interference, using two independent *CCNY* (Y #1/2) or scrambled control siRNAs at the indicated concentrations. Knockdown efficiency across all experiments, as determined by cyclin Y immunoblot, was approximately 50-70% for 293T cells (**a**) and 60-70% for HCT116 cells (**b**). Tubulin was used as loading control.
